# For Tympanites

**Published:** 1890-08

**Authors:** 


FOR TYMPANITES.—The following formula for tympanites is quoted in the *Deutsche medicinische Wochenschrift :*

| | |
|---|---|
| ℞—Naphthol | } aa 75 grains. |
| Magnesium carbonate | |
| Pulverized charcoal | |
| Oil of Peppermint | 10 drops. M. |

Divide into fifteen powders, of which one is to be given when required.—*Medical News.*

